# A Dedicated Tool for Presurgical Mapping of Brain Tumors and Mixed-Reality Navigation During Neurosurgery

**DOI:** 10.1007/s10278-022-00609-8

**Published:** 2022-03-01

**Authors:** Piero Chiacchiaretta, Mauro Gianni Perrucci, Massimo Caulo, Riccardo Navarra, Gaia Baldiraghi, Davide Rolandi, Sabino Luzzi, Mattia Del Maestro, Renato Galzio, Antonio Ferretti

**Affiliations:** 1 Department of Psychological, Health and Territory Sciences, University “G. d’Annunzio” of Chieti–Pescara, Via Luigi Polacchi, 11, 66100 Chieti, Italy; 2 Advanced Computing Core, Center for Advanced Studies and Technology (CAST), University “G. d’Annunzio” of Chieti–Pescara, Via Luigi Polacchi, 11, 66100 Chieti, Italy; 3 Department of Neuroscience, Imaging and Clinical Sciences, University “G. d’Annunzio” of Chieti-Pescara, Via Luigi Polacchi, 11, 66100 Chieti, Italy; 4 SerVE Srl, Via Falcone e Borsellino, 26, 65129 Pescara, Italy; 5 Department of Clinical-Surgical, Diagnostic and Pediatric Sciences, University of Pavia - Strada Nuova, 65, 27100 Pavia, Italy; 6 Maria Cecilia Hospital, Via Corriera, 1, 48033 Cotignola, RA Italy

**Keywords:** Mixed reality, Medical image, Neurosugery, MRI, Brain tumors

## Abstract

Brain tumor surgery requires a delicate tradeoff between complete removal of neoplastic tissue while minimizing loss of brain function. Functional magnetic resonance imaging (fMRI) and diffusion tensor imaging (DTI) have emerged as valuable tools for non-invasive assessment of human brain function and are now used to determine brain regions that should be spared to prevent functional impairment after surgery. However, image analysis requires different software packages, mainly developed for research purposes and often difficult to use in a clinical setting, preventing large-scale diffusion of presurgical mapping. We developed a specialized software able to implement an automatic analysis of multimodal MRI presurgical mapping in a single application and to transfer the results to the neuronavigator. Moreover, the imaging results are integrated in a commercially available wearable device using an optimized mixed-reality approach, automatically anchoring 3-dimensional holograms obtained from MRI with the physical head of the patient. This will allow the surgeon to virtually explore deeper tissue layers highlighting critical brain structures that need to be preserved, while retaining the natural oculo-manual coordination. The enhanced ergonomics of this procedure will significantly improve accuracy and safety of the surgery, with large expected benefits for health care systems and related industrial investors.

## Introduction

The brain tumor surgery presents complex challenges due to a delicate tradeoff between removing as much neoplastic tissue as possible and minimizing the loss of brain function. This is usually obtained using intraoperative direct cortical stimulation (DCS) which is considered the gold standard technique for functional mapping of the cortex [1, 2]. Nevertheless, DCS may trigger seizures and can only be performed intraoperatively, thus extending the duration of the procedure and preventing detailed preplanning of the intervention. In the last two decades, functional magnetic resonance imaging (fMRI) has emerged as a valuable tool for non-invasive assessment of human brain activity and is now used to determine brain regions that should be spared to prevent functional impairment after surgery [3–7]. In this regard, fMRI, complemented with other MRI modalities such as time of flight (TOF) or contrast-enhanced MR-angiography and diffusion tensor imaging (DTI) white matter tractography, offers relevant information on the anatomo-functional organization of eloquent cortical areas (e.g., sensorimotor, language, visual) and cortical/subcortical connections near or inside a tumor [4]. This large amount of data allows a unique opportunity to optimize treatment planning with a significant reduction of surgical time. The imaging results are usually sent to a neuronavigation system that calculates a transformation matrix between the surgical space (the “real” space) and the preoperative image space. This transformation allows the surgeon to see on a workstation screen the virtual counterpart of the tip of a pointer placed on a particular anatomical structure, overlaid on the corresponding region in the pre-acquired MRI (or CT) brain scans. In this way, neuronavigation supports the surgeon during surgery, allowing increased accuracy in identifying both target regions (the tumor) and key functional structures that need to be preserved.

However, despite that the scientific bases of these imaging techniques and the ensuing assisted surgery are well established, there are two major technological limitations in its current implementation.

Firstly, a specialized software that calculates, integrates, and promptly outputs the imaging results to surgical navigation systems is still lacking. Indeed, a complex cascade of operations is typically required to transfer, analyze, reformat, and output the data to a neuronavigator. In most cases, the analysis is carried out using packages developed for research purposes in the field of neuroimaging (AFNI, FSL, SPM, BrainVoyager). While these programs have reached a considerable level of complexity and performance, their use is not straightforward in a clinical setting. Moreover, each software is generally optimized for a specific imaging modality, so that the mastery of different packages is required to accomplish the entire procedure. As a matter of fact, presurgical mapping is still restricted to imaging centers that can count on a multidisciplinary research team in addition to a clinical team, preventing large-scale diffusion of presurgical mapping.

Secondly, despite neuronavigation systems offer recognized advantages in assisting the surgeon, its current implementation has limited ergonomics, forcing the neurosurgeon to continuously switch his gaze from the surgical field into a workstation screen where 2-dimensional (2D) representation of preoperative images and virtual surgical instruments is shown. This requires to the surgeon a significant effort to mentally integrate the information from different modalities and reconstruct 3-dimensional (3D) features of the patient anatomy of interest, with prolonged intervention duration and increased error risk.

In this regard, emerging techniques such as augmented reality (AR) and mixed reality (MR) are showing promising results for neurosurgical applications [8–13]. In particular, the MR approach has the largest potential in terms of increased ergonomics since it mixes real and virtual objects producing a visualization environment where physical and digital objects co-exist and interact in real time. However, previous studies mostly used anatomical images, while the full potential of functional presurgical mapping data in combination with AR or MR has not been exploited yet.

These issues motivated the main objectives of this work that are:

1. To develop a specialized software able to implement the analysis of multimodal MRI in a single application and to transfer the results to the neuronavigator, minimizing manual procedures. This will extend the diffusion of presurgical mapping procedures also to hospitals outside the academic community.
2. To develop a specialized application that integrates the full potential of presurgical mapping MRI data in a commercially available wearable device, using the emerging mixed-reality imaging technology. In particular, 3D holograms obtained from MRI will be anchored with the physical head of the patient, allowing the surgeon to easily locate the best skull area for craniotomy and, at the same time, virtually explore deeper tissue layers highlighting critical brain structures that need to be preserved. This is expected to significantly improve the surgeon accuracy and decrease mistakes.

## Methods

### Analysis Platform 

For the first objective of this work, we implemented a software platform for the analysis and integration of imaging data from different modalities useful for presurgical mapping of brain tumors, including multimodal magnetic resonance imaging (MRI) and computed tomography (CT) images. The image analysis is based on different open source software tools that have gained a solid reputation in the neuroimaging scientific community. These programs include AFNI (https://afni.nimh.nih.gov), FreeSurfer (http://surfer.nmr.mgh.harvard.edu), FSL (https://fsl.fmrib.ox.ac.uk/fsl/fslwiki/FSL), and custom-written software implemented in Python (http://www.python.org).

The platform has been implemented in a dedicated workstation and has been configured as a DICOM (Digital Imaging and Communications in Medicine) node, so that at the end of the MRI exam the images can be sent directly to the platform (in DICOM format). Upon arrival, the DICOM data are organized according to the BIDS structure (bids.neuroimaging.io), to standardize the preprocessing procedures.

The brain presurgical imaging techniques currently processed by our platform include structural MRI, fMRI, DTI, and TOF and are described below together with the related analysis procedures.

**Fig. 1:**
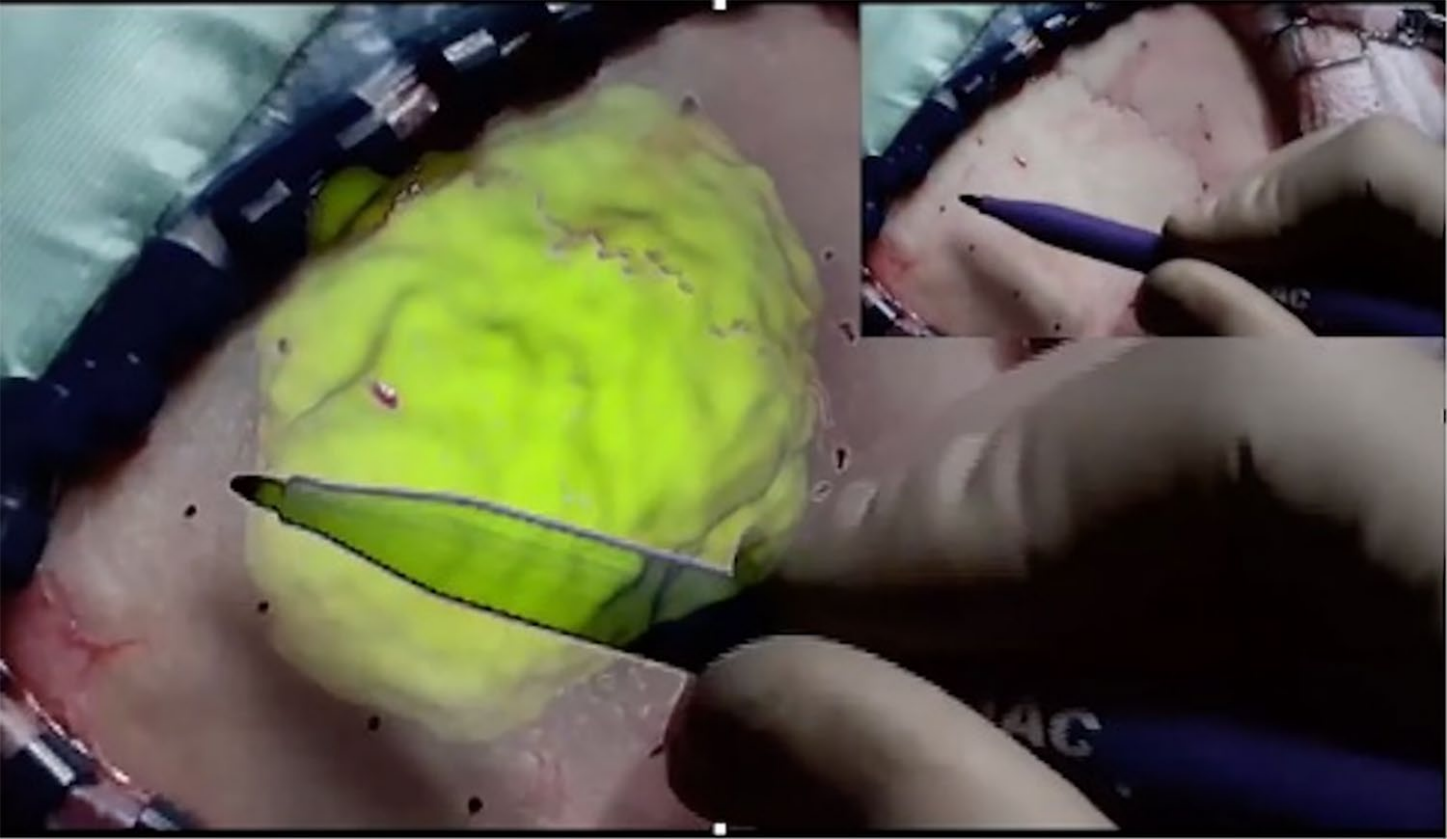
An example of a tumor hologram derived from MRI data and visualized in the HoloLens together with the “real world” scalp, after the anchorage procedure. The mixed-reality approach allows an immediate and clear understanding of the tumor position underneath the skull, helping the surgeon to delineate the best area for craniotomy

1. *Structural MRI:* These images can be obtained with different acquisition schemes yielding different types of contrasts between brain tissues. The most common MRI acquisition used for this purpose is a T1-weighted sequence yielding a good description of the shape, size, and integrity of gray and white matter structures. Moreover, structural MRI provides an anatomical reference for the visualization of the activation patterns derived from fMRI or the white matter fiber bundles derived from DTI, after a coregistration matrix between modalities has been determined. The processing of T1 images is performed using Freesurfer and includes removal of nonbrain tissue (fat, skull, or neck) and gray matter segmentation. Furthermore, 3-dimensional (3D) reconstruction of the head skin and cortical surface is accomplished using 3D Slicer and Afni routines to create meshes stored in.stl format. Tumor segmentation is also performed semi-automatically via active contour segmentation using ITK-SNAP (http://www.itksnap.org) and the corresponding mesh is saved in a.stl file as well. The head skin mesh will be finally imported in the mixed-reality application and used together with images from the HoloLens camera to perform the anchorage between virtual and real world (see later). The tumor mesh will be imported as well to visualize the corresponding hologram correctly superimposed on the patient head, allowing to highlight the lesion borders and position underneath the skin. This will help the surgeon to decide the best area for craniotomy (Fig. 1)*.*

2. Functional MRI (fMRI): Noninvasive functional mapping of the brain is usually performed using the blood oxygen level dependent (BOLD) technique [14, 15]. Briefly, a temporal series of images sensitive to the variation of deoxygenated hemoglobin occurring with neuronal activation is acquired while the patient is engaged in the execution of paradigms designed to activate eloquent areas (e.g., motor, sensorial, or language areas). For our demonstration, we used a series of T2* weighted echoplanar images (EPI) with the following parameters: TR/TE = 1000/30 ms; voxel size = 3 × 3 × 3 mm^3^; 48 slices; number of frames = 290. During fMRI, the patient was engaged in motor, visual, and language tasks performed according to a block paradigm. The preprocessing steps of fMRI data include motion correction, slice-time correction, and temporal filtering, while statistical analysis of timeseries is performed using a general linear model (GLM) with a two-gamma hemodynamic response function [16]. Coregistration between functional and structural dataset is then performed with an affine transformation.

**Fig. 2:**
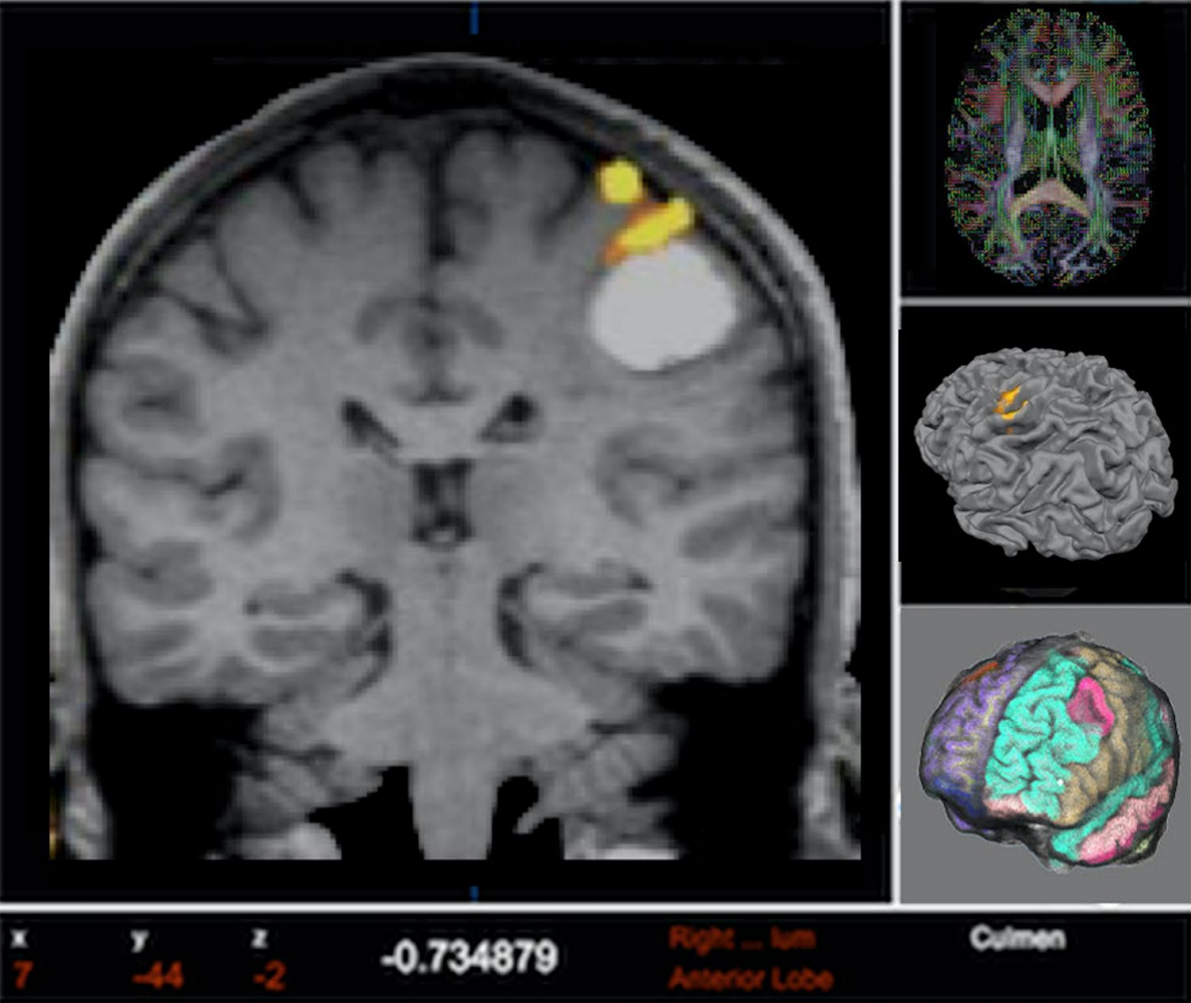
Example of fMRI activation map of the hand motor areas (red-yellow) superimposed on the structural scan (gray) of a patient with a brain tumor, as calculated by our platform

All these analysis steps have been implemented in the platform using AFNI routines. The final result is a 3D “activation map” representing eloquent cortical areas that should be spared during the surgery (Fig. 2).

**Fig. 3:**
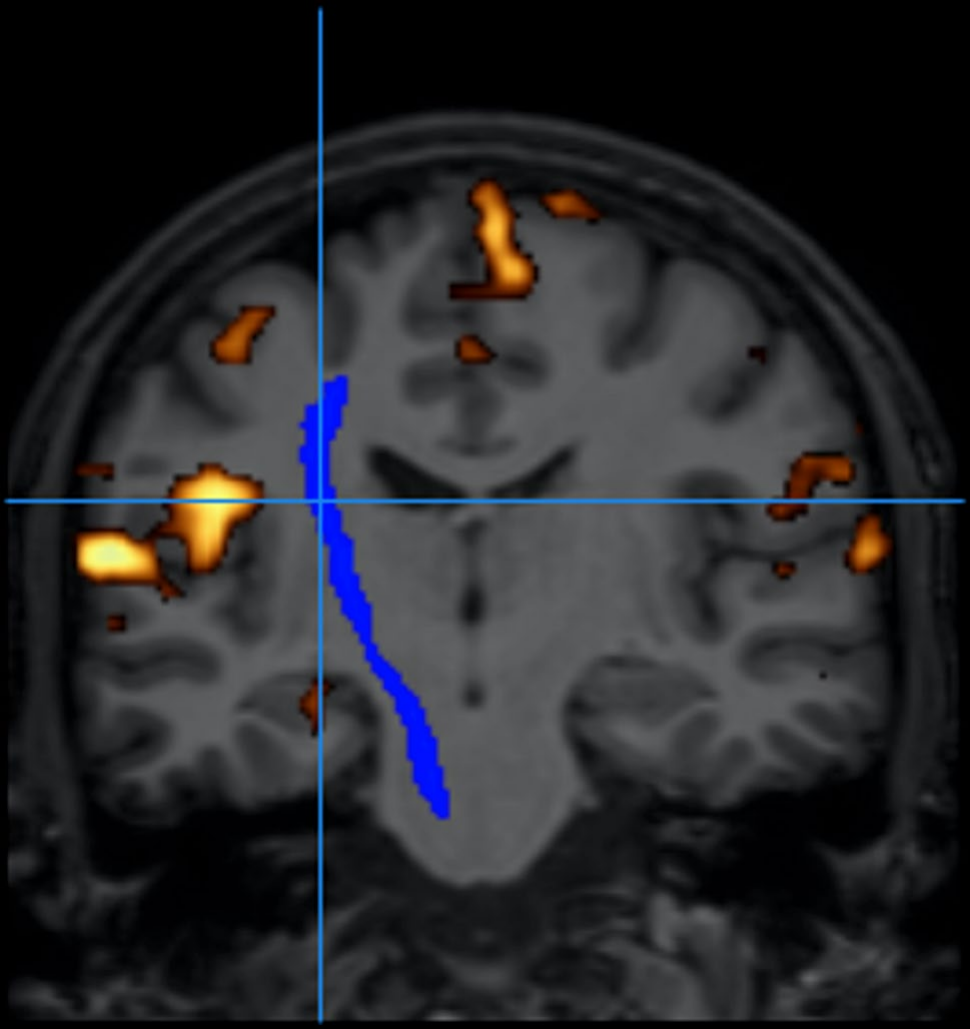
Example of the results obtained from the DTI module of our platform, showing the corticospinal tract

3. Fiber tracking (tractography): The reconstruction of white matter fiber bundles connecting different functional regions is obtained using diffusion tensor imaging (DTI) data [17]. In this MRI application, images are acquired with diffusion encoding gradients applied in different directions, allowing the calculation of the diffusion tensor of water molecules in the brain tissues for each voxel in the image. The fibers direction is indicated by the tensor main eigenvector. This vector is then color-coded to yield a map of the fibers direction that is coregistered to the structural images to correctly represent the fibers architecture with respect to the tumor. This is a valuable information when deciding the safest path to reach the lesion to be removed without damaging these connections [18, 19]. DTI analysis has been implemented using FSL/Freesurfer modules to create the probabilistic tractography (Fig. 3).

4. MR angiography (MRA): Images representing the network of arterial and venous blood vessels in the brain are obtained through acquisition sequences sensitive to blood movement such as 3D time of flight (TOF) and 3D phase-contrast angiography (PCA). For this project, we used TOF acquisitions and implemented the coregistration of these images with the structural reference with algorithms derived from iterative closest point methods. These images are not routinely used in brain presurgical mapping procedures; however, we implemented this possibility for future developments.

Importantly, a user-friendly graphical interface was also designed, specifically oriented to clinical users that need straightforward tools to accomplish the procedure.

This environment virtually requires just a few steps to the operator, including (i) to start the analysis with a single command, (ii) to perform a visual check of the correct coregistration between the different image modalities, and (iii) to accomplish the semiautomatic tumor segmentation procedure.

Specifically, the complex cascade of operations needed to analyze presurgical mapping data is started automatically and contextually with the loading of the MRI exam from a Picture Archiving and Communication System (PACS). The results are also automatically prepared to be sent to the neuronavigator system and the Microsoft HoloLens.

The tumor segmentation is the only procedure that needs a little manual operation by the radiologist.

Once preprocessed, the images are shown on the platform through a webviewer (Mango) where the user can investigate the mapping results, check the between modalities coregistration, and globally evaluate the exam. Once the control process is finished, an STL file containing the mesh from each modality is created.

The platform runs on a FUJITSU CELSIUS workstation (R970 2 10 Core Xeon Silver 4114, 96 GB DDR4 RAM, ASUS TURBO-GTX1070TI-8G video card). The software architecture has been developed to be easily adapted for a cloud application that has a number of advantages for the final user, avoiding spending on hardware and the necessity to install and maintain all the needed (open source) software tools. This also greatly simplifies upgrade operations that will not be demanded to the user.

**Fig. 4:**
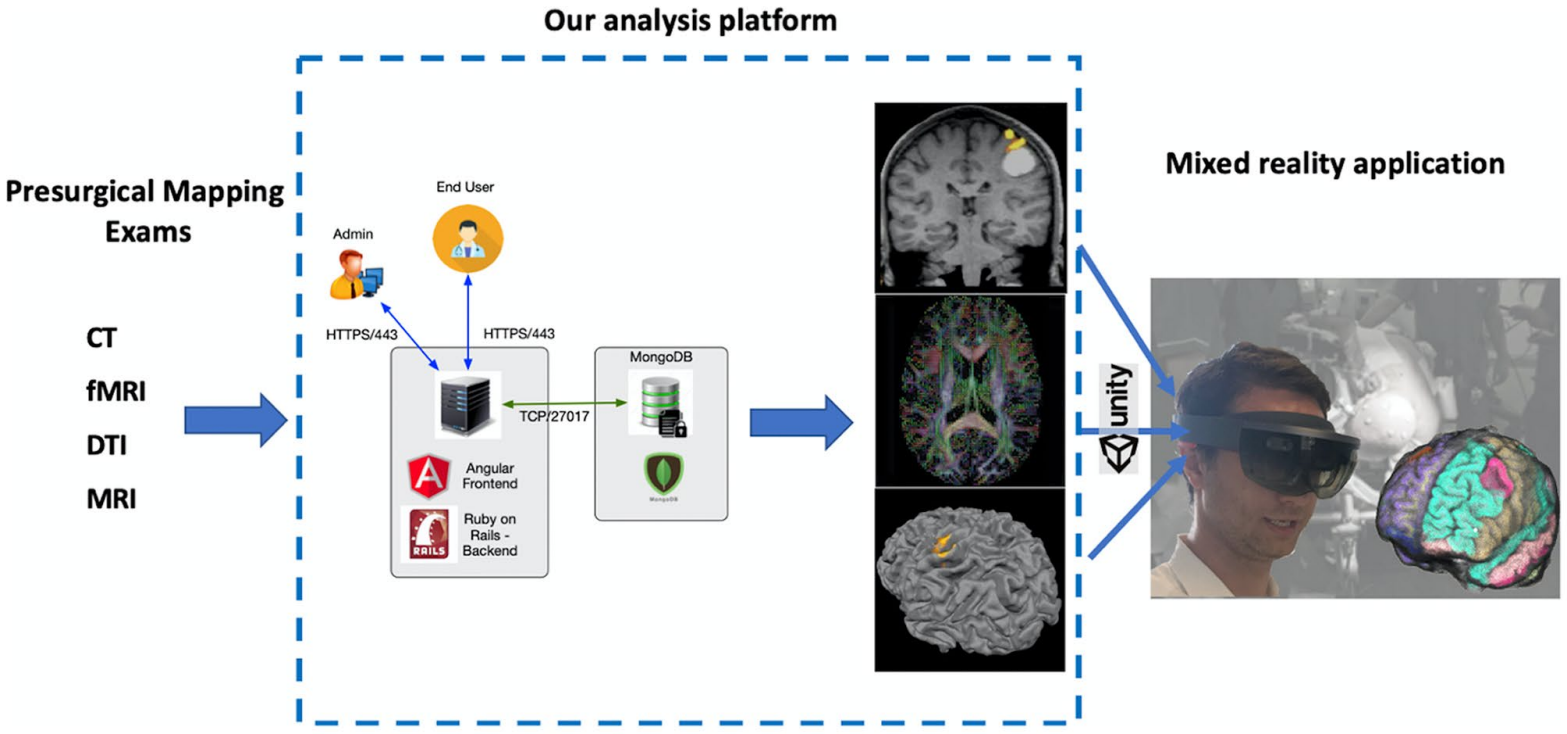
Schematic representation of our prototype analysis platform and mixed-reality application

The prototype has been implemented in a 3-tier infrastructure (Fig. 4):

- Public layer: Frontend Angular.
- Application layer: Backend Ruby on Rails.
- Database layer: MongoDB.

The authentication between the frontend and the backend is done through JavaWebToken. The frontend can be published through HTTPS using a valid certificate. The 3-tier structure of the solution allows the implementation of a network segregation assuring that the data could be stored in a most secure environment not accessible by external networks. The implementation of the frontend/backend is done through the exchange of JavaScript Object Notation (JSON) Documents. This solution increases the scalability of the technology, optimizing the implementation of future features such as a Mobile app, because the backend can be shared with Mobile and Desktop client.

Since the results are often saved in a proprietary format by the different open source tools, dedicated modules handle internally the different image formats, generally starting with the conversion from the DICOM standard (the scanner output) to the Neuroimaging Informatics Technology Initiative (NIFTI) format which is the most common input for various open-source software routines currently available for specific analyses.

**Fig. 5:**
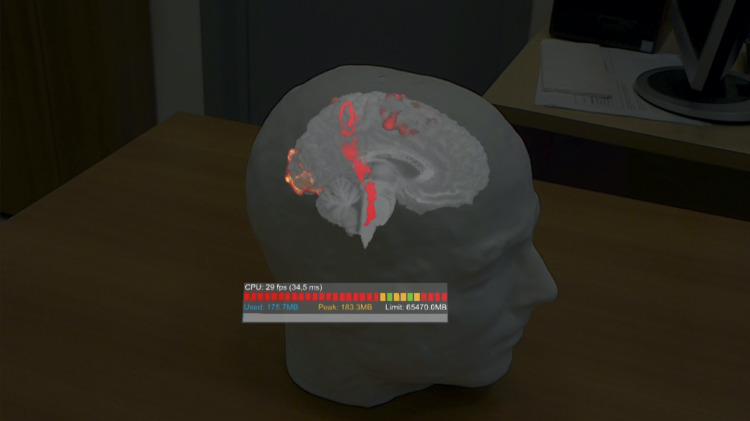
Example of the volume viewer implemented in our mixed-reality application. While the surgeon is looking at the head through the HoloLens, he can activate a reslicing option using mid-air gestures allowing to visualize internal sections obtained from volumetric structural (here in gray) and functional/DTI (here in color) data

After image processing, the mapping results are again transformed in DICOM format which is the standard accepted by current neuronavigation systems, or they are left in NIFTI to be used in the volume viewer developed for the HoloLens (Fig. 5).

## Integration of the Presurgical Mapping in the HoloLens

For the second objective of this work, we developed an application able to navigate through the presurgical mapping results obtained by our platform, using a MR approach implemented in a commercially available wearable device (Microsoft HoloLens 1.0). The application has been developed using the Unity graphical package (https://unity.com, Requirements Unity 2019.2.12f1, MRTK v2) and is able to visualize three-dimensional (3D) volumetric data (obtained from e.g. MRI and CT scans) as 3D holograms anchored with the real head of the patient (Fig. 1). Furthermore, using simple mid-air handle gestures as commands, the surgeon can reslice the volumetric images according to a preferred angulation, thus scrolling through 2D slices to view internal sections highlighting deep critical brain structures (Fig. 5). A detailed description of the application and its development is given below.

### Anchorage to the Real Head 

**Fig. 6:**
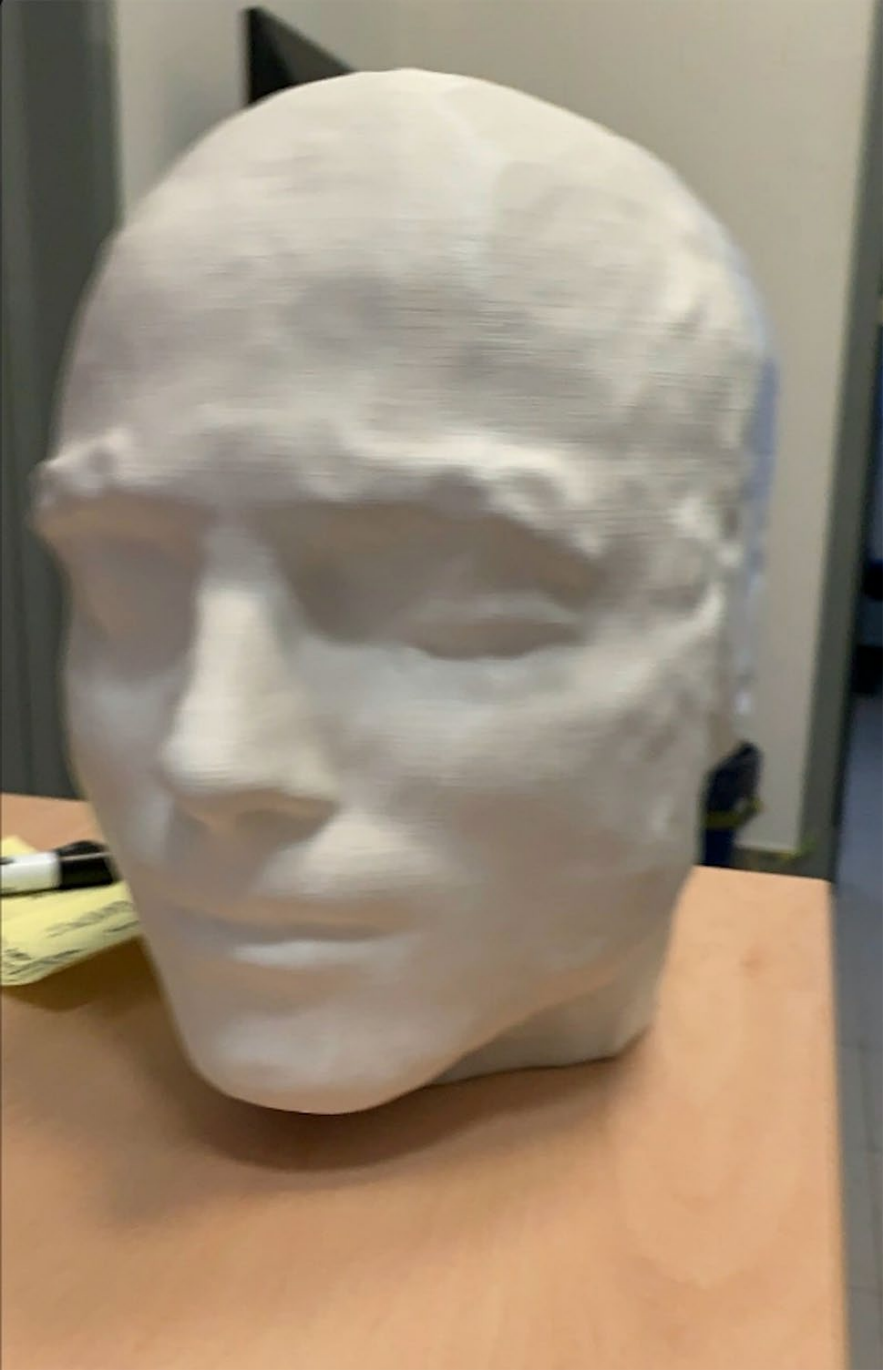
The 3D-printed head phantom obtained from a structural MRI dataset, used to develop the anchorage procedure

First, an automated anchorage procedure between the head and the virtual information (the different presurgical mapping images) has been implemented. For practical reasons, the anchorage procedure has been developed working on a head phantom realized with a 3D printer from the high-resolution anatomical MRI scan acquired on a volunteer. Specifically, a 3D virtual model of the head was created with 3dSlicer and saved in an.stl file. Starting from this file, a tangible phantom made of acrylonitrile butadiene styrene (ABS) was realized using a 3d printer (Leonardo Revo, MTC3D, https://www.mtc3d.com) (Fig. 6).

Then, we used the software development kit (SDK) of the HoloLens to access the real world images obtained from the frontal camera of the HoloLens. Exploiting the HoloLens self-localization and spatial mapping capabilities, these images are converted into meshes that are continuously updated as the user moves around the phantom. These meshes and the mesh imported from the (MRI-derived) 3D virtual model of the head are then available as input for computer-vision algorithms to accomplish the anchorage between virtual objects and the real world. Specifically, we used the algorithms available in the Vuforia library (https://developer.vuforia.com) and worked in the Unity environment to implement our anchorage procedure.

Note that the anchorage obtained using the head skin also holds for all the other MRI meshes and 3D volumes (in particular functional and tractography maps), since the between modalities coregistration has already been accomplished by the analysis platform.

### The Overlay Volume Viewer

In the developed application, we also implemented a volume viewer that allows to reslice volumetric MRI data according to a preferred direction (Fig. 5). This gives to the surgeon additional information with respect to the holographic rendering of the head, allowing a precise understanding of anatomical and functional structures located deeper in the brain. The visualization options include the possibility to (i) select the MRI volume to reslice/visualize (structural, fMRI, DTI, etc.); (ii) select the reslicing plane; (iii) overlay fMRI and DTI data on the structural scan; and (iv) change the statistical threshold of the fMRI maps (increasing the threshold allows to better locate the peaks of activation).

These features have been implemented in Unity using the ray-cast (shader) routines. In addition, a custom function allowing multiple overlays was developed. Importantly, all the visualization options can be selected by the user via mid-air gestures that helps keeping the whole procedure more sterile, allowing the neurosurgeon to consult the data in a simple and fast way.

## Results

Both the analysis platform and the MR application were evaluated by the clinical staff involved in the project. The radiologists indicated that the platform offered a significant improvement to the overall analysis procedure in terms of automation and ease of use of the graphical interface with respect to existing software packages. Also, the absence of installation and maintenance issues was greatly appreciated. The neurosurgeons indicated that the implemented MR solution is very useful for surgery planning. In particular, the overlay VolumeViewer, allowing the possibility to scroll through and simultaneously visualize the holograms corresponding to fMRI, DTI, and structural images, received a very positive feedback. Furthermore, the speed and ease of the automatic anchorage procedure was very appreciated. Finally, the surgeons suggested a great potential of the general system not only for surgery planning but for intraoperative surgery guidance as well, also considering the sterile setup offered by the mid-air gesture commands.

## Discussion

In this paper, we describe the implementation of (i) a “clinical” software platform to analyze data obtained from multimodal presurgical mapping of brain tumors and (ii) an application that integrates the mapping results in a commercially available wearable device using an optimized mixed-reality approach, offering immersive and increased visualization capabilities.

Despite that advanced structural and functional non-invasive MRI techniques, including fMRI and DTI tractography, have been increasingly used in highly specialized centers as presurgical mapping procedures helping the surgeons to preserve brain function while obtaining maximal tumor resection, their diffusion outside the academic community is still limited due to the complexities of the analysis tools. We emphasize that, to our best knowledge, a specialized and integrated software for the analysis of presurgical mapping data is still lacking.

In this regard, our solution offers an elevated level of automation and a user-friendly graphical interface specifically designed to release the clinical user from the typical complexities of research tools, thus allowing even nonspecialists in image processing to accomplish the procedure. Moreover, we based the implementation on a cloud approach to increase the platform scalability and to release the user from further issues regarding installation and maintenance operations. Although the analysis steps are based on different open source neuroimaging tools, the user is not required to master any of these packages and can remain focused on the interpretation of the results. The platform requires minimal manual actions in order to be significantly convenient in a clinical setting. In particular, our platform is executable by a single operator that, in addition to the semiautomatic tumor segmentation, should only perform a final visual check (e.g., on the correct coregistration between the functional and anatomical images) before enabling the output to the available neuronavigation systems.

We also highlight the limitations of standard neuronavigation devices. In these systems, a 2D virtual environment is created into a workstation screen including virtual surgical instruments and patient-specific virtual anatomic details obtained from pre-operative CT or MRI scans. The surgeon places a pointer on a real anatomical target and observes its correspondence with the virtual one that contains additional information (e.g., eloquent cortical areas and white matter fibers that are obviously not visible on the exposed brain). Then, he has to “mentally” integrate the 2D information to get a 3D spatial relationship between the tumor and surrounding critical structures in order to choose the best surgical strategy. Even recent approaches using augmented-reality to enrich data from the real environment with virtual information have shown limited improvement in surgical neuronavigation [6]. Indeed, despite that such systems can merge images from the real world with virtual objects (e.g., preoperative images or other clinical data of the patient), the visualization is still performed on 2D devices such as monitors and tablets. Instead, in the emerging mixed-reality technique, the virtual information is “anchored” to the real object and not simply shown together. In our application, once the anchorage has been performed correctly, the overlay between the real object and the corresponding 3D hologram is automatically updated as the surgeon changes position and looks at the patient’s head from different directions, allowing an immediate and improved understanding of the spatial relationship between tumor, eloquent areas, white matter fibers, blood vessels, and skull. This feature greatly facilitates the planning of both craniotomy and surgical resection, especially when complex anatomical structures are involved, offering a much larger room for improvements in the neurosurgery field.

The increased ergonomics and the clinical applicability of head-mounted MR devices for preoperative neurosurgical planning, as compared to standard neuronavigation systems, have been demonstrated in previous studies during live and simulated surgeries.

In particular, a prospective clinical study provided a proof of concept of the clinical applicability of the HoloLens for brain tumor preoperative planning, showing significant benefits in terms of focus and attention preservation and an enhanced understanding of the spatial tumor-brain/skull relationship due to the 3D holographic visualization [10]. Furthermore, the authors obtained quantitative outcome measures showing a median deviation of 4 mm in tumor localization between a standard neuronavigation system and the HoloLens and that in 9 out of 25 patients the localization did not differ, suggesting a potential for this MR system in the operating room. In that study, a 3D hologram reconstruction of the tumor, obtained from segmentation of contrast-enhanced T1-weighted MRI structural images, was used as preoperative virtual object.

Another study increased the visualization components in the HoloLens, including the possibility to scroll through 3D MRI/CT preoperative volumetric data and intraoperative images obtained from confocal laser endomicroscopy and ultrasound, that can all be seen in virtual 2D screens inside the HMD [11]. The authors also added new visualization options allowing e.g. to adjust the scale, orientation, and transparency of virtual objects representing 3D organ models such as tumors and vascular structures that have been segmented preoperatively. Furthermore, they conducted a pilot user study asking a group of surgeons with different medical specializations (4 out of 9 were neurosurgeons) to answer specific questionnaires on the usability of the proposed system in the operating room. The results showed great appreciation for the system, especially for the possibility to visualize the selected 3D organ model as a hologram, and a general strong interest to use a HMD with MR approach for intraoperative surgical guidance in addition to the preoperative planning phase. However, in these previous works, only a specific modality of presurgical mapping data was used (mostly just the structural images showing the brain tumor). In this regard, our platform and MR application expand the state of the art by including the full potential of advanced functional presurgical mapping modalities for brain tumor surgery. In particular, fMRI of eloquent areas and DTI tractography are of paramount importance for neurosurgery and highly valued by surgeons. The inclusion of these advanced MRI techniques determines several advantages during surgery, such as tailored craniotomy, informed selection of DCS sites resulting in a decreased number of stimulations, a reduced likelihood of intraoperative seizures, and faster procedural time with reduced patient’s fatigue [4].

Nevertheless, these advanced functional imaging techniques have been so far exploited only with standard neuronavigation systems. To our best knowledge, this is the first work that integrates all the available presurgical mapping MRI modalities in a MR HMD. Our prototype allows to visualize a chosen combination of the holograms representing the brain anatomy, the tumor borders, the eloquent cortical areas, the white matter fiber bundles, and the blood vessel network, with the possibility to vary the transparency of the different objects. Furthermore, the surgeon can reslice the volumetric images according to a preferred angulation, thus scrolling through 2D slices to view internal sections highlighting deep critical brain structures, such as white matter fiber bundles and their spatial relationship with the tumor. Importantly, these functionalities can be activated using simple mid-air handle gestures as commands, without physically touching any object, which is an important asset to keep everything sterile in the operating room. Moreover, as a significant improvement, we implemented an automatic real-to-virtual anchorage procedure that will result in reduced intervention time.

### Study Limitations

In this work, we did not perform quantitative outcome measures about the accuracy of our automatic registration versus the manual registration approach used in previous studies, since at this stage we were mainly concerned with improving the automation and ergonomics of the general workflow from the analysis of presurgical mapping data to their visualization in the HoloLens. While speed is certainly important in the operating room, we understand that in order to use these systems not only for surgery planning but also to guide the intervention, accuracy needs to be assessed and in future developments we plan to do this using approaches similar to that of [10]. However, we also point out that a major issue of neuronavigation during surgery is represented by brain deformation after craniotomy. This phenomenon is known as “brain shift” and involves non-rigid deformation of brain structures due to loss of cerebrospinal fluid, changes in intracranial pressure, and other reasons. The result is that, when brain shift is significant, preoperative images do not match with images of the patient seen through the surgical microscope or HMDs. This topic is the subject of intense research [20] and we plan to work in this direction as well in future studies. Furthermore, a significant improvement in future MR technology for surgery will be the development of a dedicated HMD able to provide some magnification factor, ideally integrating the neuronavigator and surgical microscope in a single instrument with the added potential of holographic visualization.

Finally, we did not perform a user study on an extended group of neurosurgeons using structured questionnaires. However, the feedback of the clinical staff in our unit has been very positive on the usability of both the analysis platform and the MR application. In particular, the radiological staff greatly appreciated the simple workflow of the analysis procedure offered by our solution, whereas the surgeons highly evaluated the increased understanding of the spatial relationships between brain structures and the general ergonomics of the MR application.

In conclusion, despite that MR in the operating room is still at an early stage, the results from our work and previous studies provide evidence of a great potential to assist surgeons intraoperatively.

## Data Availability

Yes, upon request.

## Code Availability

Yes, upon request.
