# Correction: A Dedicated Tool for Presurgical Mapping of Brain Tumors and Mixed‐Reality Navigation During Neurosurgery

**DOI:** 10.1007/s10278-022-00750-4

**Published:** 2022-12-13

**Authors:** Piero Chiacchiaretta, Mauro Gianni Perrucci, Massimo Caulo, Riccardo Navarra, Gaia Baldiraghi, Davide Rolandi, Sabino Luzzi, Mattia Del Maestro, Renato Galzio, Antonio Ferretti

**Affiliations:** 1 Department of Psychological, Health and Territory Sciences, University “G. d’Annunzio” of Chieti–Pescara, Via Luigi Polacchi, 11, 66100 Chieti, Italy; 2 Advanced Computing Core, Center for Advanced Studies and Technology (CAST), University “G. d’Annunzio”of Chieti–Pescara, Via Luigi Polacchi, 11, 66100 Chieti, Italy; 3 Department of Neuroscience, Imaging and Clinical Sciences, University “G. d’Annunzio” of Chieti-Pescara, Via Luigi Polacchi, 11, 66100 Chieti, Italy; 4 SerVE Srl, Via Falcone e Borsellino, 26, 65129 Pescara, Italy; 5 Department of Clinical-Surgical, Diagnostic and Pediatric Sciences, University of Pavia - Strada Nuova, 65, 27100 Pavia, Italy; 6 Maria Cecilia Hospital, Via Corriera, 1, 48033 Cotignola, RA Italy

**Correction to: Journal of Digital Imaging (2022)** 10.1007/s10278-022-00609-8

In the original article, an image taken from a work of the group of Prof. D. Louis Collins (REF) has been inserted as Fig. 1 instead of the following picture from our work.
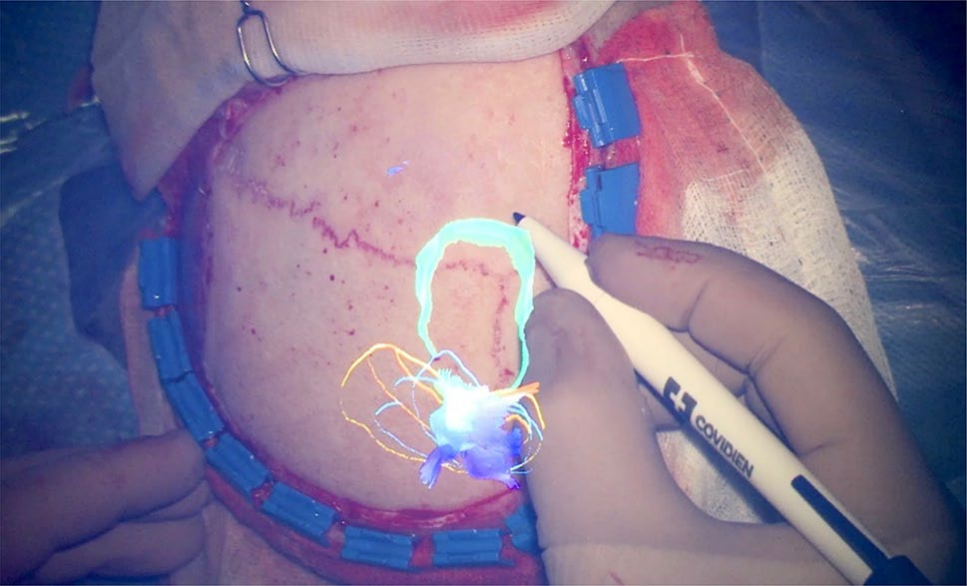


Futhermore, we would like to acknowledge additional work by Prof. Collins including the following references:

1. Drouin S, Kochanowska A, Kersten-Oertel M, et al. IBIS: an OR ready open-source platform for image-guided neurosurgery. Int J Comput Assist Radiol Surg 2017;12:363–378.
2. Drouin S, Kersten-Oertel M, Chen SJS, Collins DL. Realistic Test and Development Environment for Mixed Reality in Neurosurgery. Augmented Environments for Computer Assisted Interventions 2012: 13–23.
3. Drouin S, Di Giovanni DA, Kersten-Oertel M, Collins DL. Interaction driven enhancement of depth perception in angiographic volumes. IEEE Trans Vis Comput Graph 2018;26:2247–2257.
4. Kersten-Oertel M, Chen SS, Drouin S, Sinclair DS, Collins DL. Augmented reality visualization for guidance in neurovascular surgery. Stud Health Technol Inform 2012;173:225–229.
5. Kersten-Oertel M, Chen SJ, Collins DL. An Evaluation of Depth Enhancing Perceptual Cues for Vascular Volume Visualization in Neurosurgery. IEEE Trans Vis Comput Graph 2013.
6. Kersten-Oertel M, Gerard I, Drouin S, et al. Augmented reality in neurovascular surgery: feasibility and first uses in the operating room. Int J Comput Assist Radiol Surg 2015;10:1823–1836.
7. Kersten-Oertel M, Gerard IJ, Drouin S, et al. Augmented Reality for Specific Neurovascular Tasks. In: Linte CA, Yaniv ZR, Fallavolita P, ed. Augmented Environments in Computer Assisted Interventions; 2015; Munich LNCS
8. Gerard IJ, Kersten-Oertel M, Drouin S, et al. Combining intraoperative ultrasound brain shift correction and augmented reality visualizations: a pilot study of eight cases. Journal of medical imaging 2018;5:021,210.

Also, the sentence in the original article: “To our best knowledge, this is the first work that integrates all the available presurgical mapping MRI modalities in a MR HMD” has been changed in “To our best knowledge, this is the first work that integrates this combination of functional and structural presurgical mapping MRI modalities in a MR HMD”.

As a minor correction, the affiliation of the last author includes both centers 3 and 4.

The authors apologize for these issues and state that these modifications do not change the results or conclusions reported in the article in any way. The original article has been updated with these changes.

